# General method for classification of fiber families in fiber-reinforced materials: application to in-vivo human skin images

**DOI:** 10.1038/s41598-020-67632-z

**Published:** 2020-07-02

**Authors:** Maximilian Witte, Sören Jaspers, Horst Wenck, Michael Rübhausen, Frank Fischer

**Affiliations:** 1 Center for Free-Electron Laser Science (CFEL), University of Hamburg, 22607 Hamburg, Germany; 2 Beiersdorf AG, 20245 Hamburg, Germany

**Keywords:** Computational models, Tissues

## Abstract

Fiber structures play a major role for the function of fiber-reinforced materials such as biological tissue. An objective classification of the fiber orientations into fiber families is crucial to understand its mechanical properties. We introduce the Fiber Image Network Evaluation Algorithm (FINE algorithm) to classify and quantify the number of fiber families in scientific images. Each fiber family is characterized by an amplitude, a mean orientation, and a dispersion. A new alignment index giving the averaged fraction of aligned fibers is defined. The FINE algorithm is validated by realistic grayscale Monte-Carlo fiber images. We apply the algorithm to an in-vivo depth scan of second harmonic generation images of dermal collagen in human skin. The derived alignment index exhibits a crossover at a critical depth where two fiber families with a perpendicular orientation around the main tension line arise. This strongly suggests the presence of a transition from the papillary to the reticular dermis. Hence, the FINE algorithm provides a valuable tool for a reliable classification and a meaningful interpretation of in-vivo collagen fiber networks and general fiber reinforced materials.

## Introduction

Biological tissue such as articular cartilage^1^, myocardium^2^, aortic valve^3^, arterial walls^4^, and skin^5^ exhibit a stress strain behavior that strongly depends on the collagen fiber distribution. Fiber reinforced materials are classified by the underlying fiber network which is characterized by its anisotropy and the fiber orientation^6–8^. Upon stretching, tensile forces are applied to biological specimens and collagen fibers align in the stretching direction^9–14^. The characterization of the collagen network is typically determined by quantities like the orientation index, mean fiber orientation, and the fiber dispersion. These parameters are obtained from the angular orientation distribution which is commonly modeled by a pi-periodic von-Mises function^15–21^. However, this approach assumes that all fibers are part of a single fiber family. Gasser et al. introduced a mechanical model for arterial walls which assumes the existence of two opposing collagen fiber families, which are oriented around a main direction^4^. Parameters for this model are achieved by modeling the fiber orientation distribution using two pi-periodic von-Mises functions^22^. Skin is of major relevance as it represents the largest organ of the human body. It is subject to diverse environmental stress conditions and also large mechanical strains. Langer lines, also known as cleavage lines, are reported to indicate the main orientation of collagen fibers in skin^16^.

We introduce the Fiber Image Networks Evaluation algorithm (FINE algorithm), which is based on the cumulative orientation distribution (COD), to classify and quantify the fiber network by means of fiber families. The FINE algorithm uses an iterative approach to identify the number of fiber families and their angular properties. The variance of the COD that is obtained by the adaptive Fourier filtering method, proposed in^23^, is used to estimate the significance of each fiber family. To benchmark the FINE algorithm, realistic grayscale Monte-Carlo simulated fiber images containing multiple fiber families are used. We derive the minimum fraction of anisotropic fibers as well as the maximum number of highly aligned fiber families that the FINE algorithm is able to discriminate. In addition to the orientation index (OI), we introduce and validate the alignment index (AI) which quantifies the average alignment degree of different fiber families. We apply the FINE algorithm to in-vivo, three-dimensional images of collagen fibers in human skin. Indeed, at a depth of 85–90 µm we find an increase of the derived alignment index with a concurrent decrease of the orientation index. Furthermore, two intersecting fiber families with a perpendicular orientation around the Langer line arise. This strongly suggests the presence of a transition from the papillary to the reticular dermis.

## Results and discussion

### Fiber image network evaluation algorithm (FINE algorithm)

**Figure 1:**
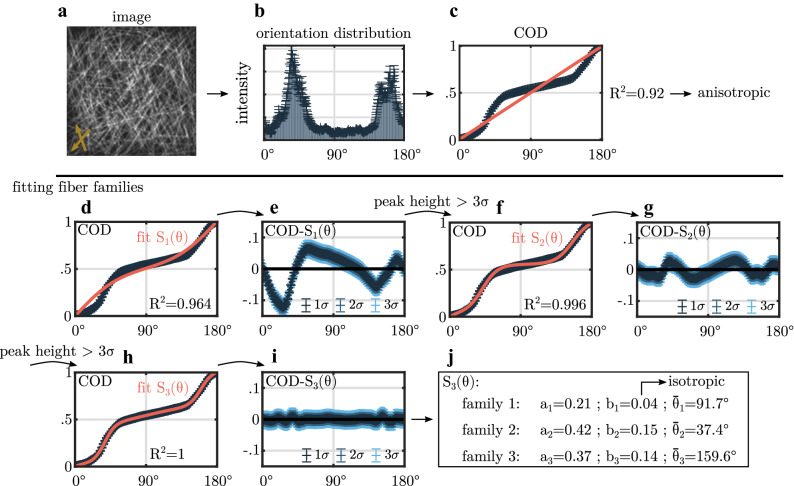
Schematic representation of the FINE algorithm. (**a**) Exemplary Monte-Carlo simulated fiber image with 300 isotropically distributed fibers and two aligned fiber families, each containing 175 fibers $({{\bar{\theta}}}_{2}={40}^\circ\quad {\text{and}}\quad {{\bar{\theta}}}_{2}={160}^\circ ;{b}_{2}={b}_{3}={0.18})$. (**b**) The angular orientation distribution $I(\theta )$ is achieved according to Witte *et al.*^23^. (**c**) As a first step the cumulative orientation distribution (COD) is checked for isotropy as the ${R}^{2}$ value of a straight line with slope ${1/180}^\circ$ is examined. (**d**) Fit of a single step function to model one fiber family (${S}_{1}$ Eq. (8)) to the COD. (**e**) Difference between the COD and ${S}_{1}$ with subsequent peak finding. (**f**) Fit of two fiber families using a series of two sigmoid functions ${(S}_{2})$. (**g**) Difference between the signal COD and ${S}_{2}$ with subsequent peak finding. (**h**) Final fit of three fiber families using a series of three sigmoid functions ${S}_{3}$. (**i**) Final difference between the COD and ${S}_{3}$. Since no significant residuals are present, the algorithm terminates. (**j**) Summary of the fit parameters of the three fiber family fit ${S}_{3}$. Two highly aligned fiber families in addition to one isotropic fiber family are identified.

We develop a general method for classification of fiber families in fiber-reinforced materials, Fiber Image Network Evaluation Algorithm (FINE algorithm). In order to explain the algorithm, we use an artificial fiber image, shown in Fig. 1(a). Artificial, grayscale fiber images are created using a Monte-Carlo procedure that allows us to control the number of fiber families, their amplitudes and their fiber distributions.

In the FINE algorithm, the number N, the mean orientations ${{\bar{\theta}}}_{i}$, the amplitudes ${a}_{i}$ and the dispersion ${b}_{i}$ of fiber families, based on the cumulative orientation distribution (COD) of an input fiber image, are identified. We obtain the COD by applying the adaptive Fourier filter method (AF method), as proposed by Witte *et al.*^23^, to a fiber image. Exemplary, this is shown for an artificial, grayscale Monte-Carlo fiber image in Fig. 1(b). The proposed AF method provides the variance σ of the COD, which we use in the FINE algorithm as termination criterion.

We use the sigmoid function of Eq. (5) to model the COD of one fiber family. This sigmoid function has a step at the mean orientation angle ${\bar{\theta}}$, a steepness given by the dispersion $b$ of the fibers around its mean orientation and a height given by the amplitude $a$. Furthermore, the analysis of $N$ fiber families in the fiber image is realized by modeling the COD as a sum of $N$ sigmoid functions [Eq. (8)]. The number $N$ of fiber families is iteratively determined by the FINE algorithm. The FINE algorithm starts with the most trivial assumption of a completely isotropic fiber distribution. Such a distribution is described by a straight line with slope 1°/180° in the COD. Thus, the first step in the algorithm is to check for an isotropic distribution by evaluating the ${R}^{2}$ value of the straight line. Exemplary, this is shown in Fig. 1(c) for the artificial Monte Carlo generated fiber image of Fig. 1(a). In Fig. 1(c), the ideal isotropic fiber distribution is represented by a red, straight line. In the FINE algorithm, a fiber distribution is considered as isotropic for a fit quality better than ${R}^{2}={0.9916}$. In our example, ${R}^{2}={0.92}$ indicates the presence of at least one fiber family, and the first sigmoid function (Eq. (8) with *N* = 1) is fitted to the COD. The resulting fit of a single fiber family is shown in Fig. 1(d). The quality of the fit increases to ${R}^{2}={0.964}$. However, the difference between the single sigmoid fit and the COD, shown in Fig. 1(e), is larger than 3σ. For the FINE algorithm, this is the criterion to consider an additional fiber family. Now, the angular location of the largest deviation between COD and fit is considered as new starting value for the additional fiber family. The corresponding fit of a series of two sigmoid functions $(N={2})$ is shown in Fig. 1(f). Although a better goodness of the fit with ${R}^{2}={0.996}$ is reached, the corresponding residual, as shown in Fig. 1(g) indicates the existence of another significant amplitude. Again, we employ the location of the highest deviation between fit and COD as initial location for a third sigmoid function $(N={3}).$ In our fiber image simulation example the COD and the fit are presented in Fig. 1(h). We now obtain ${R}^{2}={1}$, and the residual between fit and COD is within the 3σ criterion (Fig. 1(i)). This terminates the FINE algorithm.

The fit parameters received from the FINE algorithm, shown for our example in Fig. 1(j), are now used to evaluate the structure of the fiber network. In our fiber image, a small dispersion coefficient of ${b}_{1}={0.04}$ indicates that this fraction of fibers is isotropically distributed. Fiber families 2 and 3 are highly aligned with ${b}_{2}={0.15}$ and ${b}_{3}={0.14}$. The amplitudes ${a}_{i}$ are a measure for the fraction of each fiber family with respect to the whole fiber network. The amplitudes show that the aligned fiber families 2 and 3 exhibit a similar fiber fraction within the network $({a}_{2}={0.42}\ {\text{and}}\ {a}_{3}={0.37}).$ In contrast, the isotropic fiber family 1 contributes less with ${a}_{1}={0.21}$. Since we used a Monte-Carlo generated fiber image as input in our example, the determined parameters found by FINE algorithm can be compared to the preset values of the Monte Carlo simulation. For our example, we find that the dispersion coefficients of fiber families 2 and 3 calculated by the FINE algorithm underestimate the Monte-Carlo input value of $b=0.18$, indicating a broadening of the distribution of aligned fibers. Predefined mean orientations of the highly aligned fiber families with ${{\bar{\theta}}}_{2}={40}^\circ$ and ${{\bar{\theta}}}_{3}={160}^\circ$ are reproduced with ${{\bar{\theta}}}_{2}=37.4^\circ$ and ${{\bar{\theta}}}_{3}=159.6^\circ$.

In order to evaluate the FINE algorithm, we generate Monte-Carlo fiber images with systematically modified properties and compare preset fiber network parameters with the network parameters calculated by the FINE algorithm.

### FINE evaluation using Monte-Carlo images

Our general expectation to a fiber distribution includes isotropic as well as aligned parts. The quantification of both parts is crucial in order to identify an anisotropic material behavior. Since the FINE algorithm calculates the dispersion as well as the amplitude of each fiber family, we are analyzing its ability to discriminate the aligned part from the isotropic part. In order to control the aligned part of our Monte-Carlo images, we define the anisotropic ratio of fibers (ARF) of our Monte-Carlo simulated images. The ARF measures the number of fibers contributing to an aligned fiber family relative to the total number of sampled fibers.

### Anisotropic ratio of fibers

**Figure 2:**
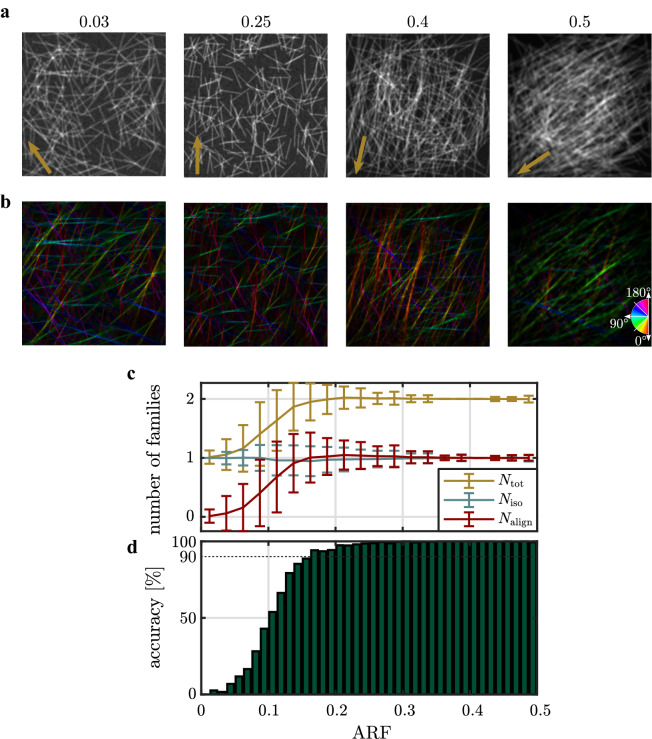
Result of the FINE algorithm as a function of the anisotropic ratio of fibers (ARF) of Monte-Carlo simulated fiber images. (**a**) Representative Monte-Carlo images with a different ARF. Mean orientations of the aligned fiber families are indicated by arrows in the bottom, left corner. (**b**) Calculated local main orientation in false colors. (**c**) Mean number of fiber families ${N}_{\text{align}}$ (aligned), ${N}_{\text{iso}}$ (isotropic) and ${N}_{\text{total}}$ (total) that were identified by the algorithm. Error bars represent the standard deviation. (**d**) Accuracy of the algorithm to identify exactly one aligned and one isotropic fiber family.

Monte-Carlo images with two fiber families, one isotropic and one highly aligned family are created. In the process, we use a constant number of 200 isotropically distributed fibers, together with a variable number of highly aligned fibers as anisotropic part. The maximum number of aligned fibers is limited to 200 (ARF = 0.5), whilst zero aligned fibers ensure a pure isotropic distribution (ARF = 0). A total of ${10}^{4}$ images are generated to guarantee a high statistical accuracy of the result. Exemplary Monte-Carlo images with a different ARF are shown in Fig. 2(a). Figure 2(b) shows the calculated local fiber orientation in false colors. Color-coded fiber angles visually coincide well with the expected angles.

For quantitative evaluation, we measure different parameters of the FINE algorithm as a function of the ARF. The mean number of total fiber families ${N}_{\text{tot}}$, the mean number of anisotropic fiber families ${N}_{\text{align}}$ and the mean number of isotropic fiber families ${N}_{\text{iso}}$ identified by the algorithm are shown in Fig. 2(c). As expected, the mean number of isotropic fiber families constantly remains at a mean value of ${N}_{\text{iso}}={1}$. Contrary, its standard deviation first increases to maximum of 0.25 at a ratio of $\text{ARF}=0.12$ and then decreases to a constant value of $\sim 0.05$ for a fraction larger than ${\text{ARF}}>0.35$. The mean number of identified aligned fiber families increases from near zero at a vanishing anisotropic part to a value of ${N}_{\text{align}}={1}$ for a ratio of ${\text{ARF}}\ge 0.163$. The determined mean orientation ${\bar{\theta}}$ of the aligned fiber family exhibits an absolute deviation to the reference angle of $\Delta {\bar{\theta}}=\left(2.4\pm 2.5\right)^\circ$. Next, we determine the probability that the FINE algorithm identifies exactly one isotropic and one highly aligned fiber family, which is shown in Fig. 2(d). The accuracy rapidly increases with the number of aligned fibers. At a ratio of $\text{ARF}=0.163$, a 90% accuracy for the identification for one isotropic and one aligned fiber family is reached. For ${\text{ARF}}\ge 0.163$, the error of the calculated mean orientation ${\bar{\theta}}$ of the aligned fiber family decreases to $\Delta {\bar{\theta}}=\left(2.0\pm 1.9\right)^\circ$. The mean amplitude of the aligned fiber family increases from near zero at a vanishing ARF to 0.6 at ${\text{ARF}}= 0.5$ (Supplementary Fig. 1(a)). Remarkably, the calculated amplitudes are overestimating the anisotropic ratio. Since grayscale images are created, where fiber intensities are added to the image, the overlay of multiple fibers causes an artifact in the angular orientation distribution^18^. Additionally, isotropic fibers that are by chance oriented in the direction of the aligned fiber family might further raise the intensity of the aligned fiber family.

Furthermore, the dispersion of the aligned fiber family constantly increases with the ARF but remains underneath the predefined value of $b=0.18$ (Supplementary Fig. 1(b)). This indicates a broadened angular width of the aligned fiber family with respect to its defined value. The deviation to the reference most likely originates from the significant angular overlap between both fiber families, such that fibers either contribute to the anisotropic or the isotropic part. A very high mean goodness of the fit of ${R}^{2}=0.999\pm 0.001$ indicates a very good representation of the COD by the FINE algorithm.

### Multiple aligned fiber families

Another important evaluation aims to capture the performance of the FINE algorithm to identify multiple non-overlapping highly aligned fiber families. To ensure a clear angular separation of each created fiber family, a minimum angular distance between adjacent families has to be enforced. In order to find a good estimate for the minimum angular distance, the ability of the algorithm to separate two equally dispersed families from each other is investigated. Supplementary Fig. 2(a) shows the mean number of identified fiber families for Monte-Carlo images with two equally dispersed fiber families as a function of their alignment and their angular distance. If the angular distributions of both fiber families are exceeding a critical angular overlap, as exemplary shown in Supplementary Fig. 2(b) and (c) the FINE algorithm identifies a single fiber family. A clear separation of both families can be accomplished by a vanishing angular overlap, which is ensured for a minimum angular distance of 30° and a high alignment of $b>0.16$ (Supplementary Fig. 2(d)).

**Figure 3:**
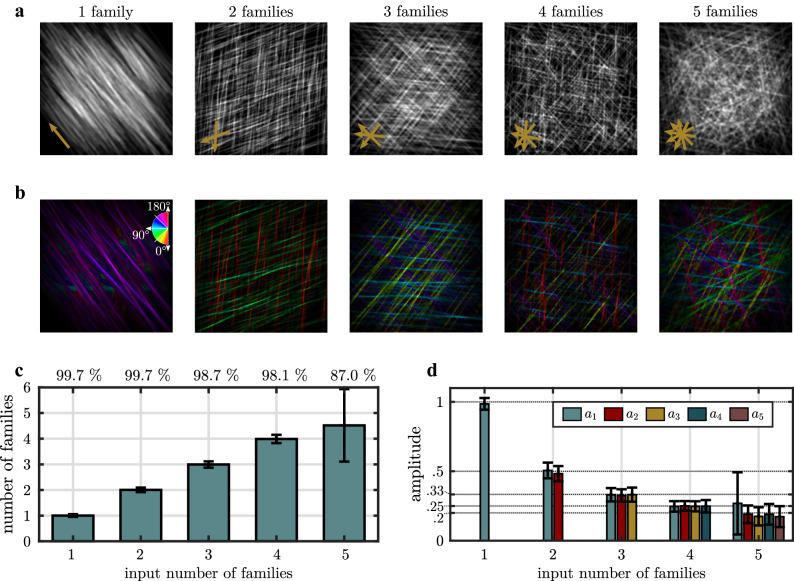
Result of the FINE algorithm applied to Monte-Carlo simulated grayscale fiber images containing one to five fiber families. (**a**) Representative images with one to five fiber families. Mean fiber orientations are indicated by arrows in the bottom, left corner. (**b**) Calculated local main orientation in false colors. (**c**) The mean number of fiber families estimated by the algorithm. Error bars represent the standard deviation. The percentage of images for which the number of estimated fiber families matches the number of defined fiber families is specified at the top. (**d**) The amplitudes of each identified fiber family. Error bars represent the standard deviation. Expected amplitude levels at 1, 0.5, 0.33, 0.2 are marked with dotted lines. Note that amplitudes below a value of 0.01 are removed for the sake of clarity.

We generate Monte-Carlo images containing up to five highly aligned fiber families, that are exemplary shown in Fig. 3(a). The number of fibers contributing to one fiber family as well as their dispersion are held constant. The calculated local fiber orientations are shown in false colors in Fig. 3(b). The COD is modeled with ${R}^{2}=0.999\pm 0.002$. Mean orientation angles of each aligned fiber family are found with a high accuracy as the absolute deviation to the reference mean orientations amounts to $\Delta {\bar{\theta}}=\left(0.7\pm 0.5\right)^\circ$. The ratio of images where the predefined number of fiber families is identified, decreases from 99.7% in case of one and two predefined fiber families down to 87.0% in case of five predefined fiber families (Fig. 3(c)). The mean number of identified fiber families matches the number of predefined fiber families for Monte-Carlo images with up to four fiber families. With an increase of fiber families, the probability to wrongfully identify the network as an isotropic network increases, since an infinite number of fiber families yields an isotropic network. The amplitude of each identified fiber family as well as expected amplitudes 1, 0.5, 0.33, 0.2 are shown in Fig. 3(d). Mean amplitudes are found to closely match the expected values.

### Dermal collagen fiber network

Collagen fibers represent the major load-bearing component of connective tissue such as the dermal skin layer^16,24,25^. Contrary to the straight fibers that are sampled in our artificial Monte-Carlo images, collagen fibers are wavy and bended^17^ Compared to the angular orientation distribution of a network of straight fibers, the angular orientation distribution of a wavy and bended fiber network is broadened. Since the FINE algorithm processes the entire angular orientation distribution of an image, the waviness of fibers does not influence the algorithms accuracy. We capture the second harmonic generation (SHG) signal of dermal collagen fibers by using multi-photon microscopy, which is a common tool to visualize collagen fibers of human skin in-vivo^26,27^. We apply the FINE algorithm to an in-vivo depth scan of the SHG signal of dermal collagen in human skin. Depths from 60 µm up to 105 µm relative to the skin surface are measured. Mean orientation angles, amplitudes and dispersions of identified fiber families are evaluated. Additionally, derived parameters, which quantify the entire orientation distribution are calculated. The orientation index (OI) describes the global alignment of the fibers with respect to their main orientation based on the angular orientation distribution^28^. Contrary, we define the alignment index (AI) as a measure of the global alignment of the fibers, independent from their main orientation. Assume $N$ identified fiber families with amplitudes ${a}_{i}$ and dispersion parameter ${b}_{i}$. The AI then reads as:

1 $${\text{AI}}=\sum_{1}^{N}{a}_{i}\cdot {b}_{i}^{\prime}\quad {\text{with}}\quad {b}_{i}^{{\prime}}=\frac{{b}_{i}-{b}_{\min}}{{b}_{\max}-{b}_{\min}}.$$

${b}_{i}^{\prime}$ represents the normalized dispersion coefficient with normalization quantities ${b}_{\min}$ and ${b}_{\max}$. For example, a fiber network of two highly aligned fiber families that are arranged perpendicular to each other exhibit an OI of zero and an AI of one. We further illustrate the difference between the OI and the AI in the Supplementary Fig. 3.

**Figure 4:**
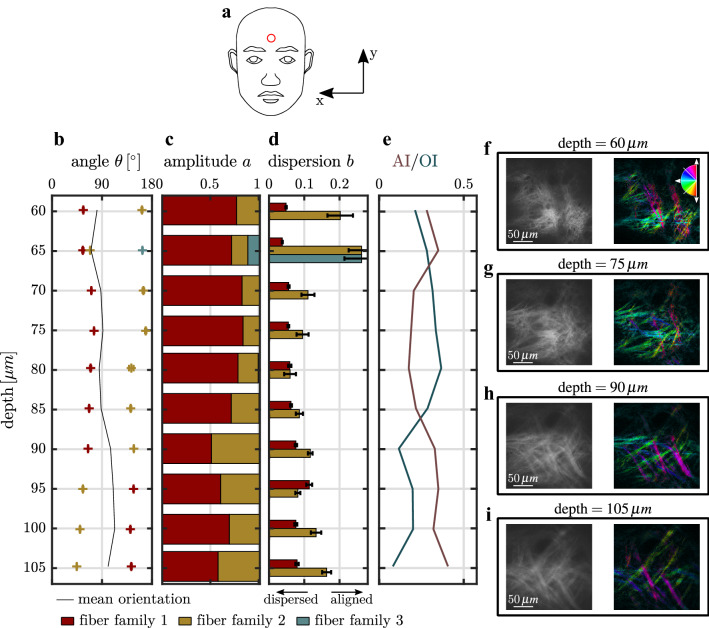
Results of the FINE algorithm of in-vivo multi-photon SHG images as a function of skin depth. (**a**) Location of the measurement and origin of the coordinate system. (**b**) Mean orientation angles ${{\bar{\theta}}}_{i}$ of the identified fiber families. (**c**) Amplitudes ${a}_{i}$ of each identified fiber family. (**d**) Dispersion parameter ${b}_{i}$ of each identified fiber family. Error bars represent the 95% confidence intervals. (**e**) Derived parameter AI (alignment index) and OI (orientation index) as a function of depth. (**f**–**i**) Exemplary SHG images (left) and the local fiber orientation shown in false colors (right). The color wheel shows the assignment of each color.

Figure 4 summarizes the result of the FINE algorithm as well as the corresponding parameters as a function of dermal depth. In general, two fiber families are identified. The weighted mean orientation of both fiber families, shown in Fig. 4(b), fluctuates around 90°, which coincides with the direction of the so called Langer lines^29^. Langer lines are the main tension lines of human skin, that were correlated to a preferred orientation of collagen in ex-vivo experiments^30–32^. At a depth of 60 µm, fiber family 1 dominates the fiber distribution with an amplitude that is 3.2 times stronger as compared to fiber family 2, shown in Fig. 4(c). With increasing depth, the amplitudes of both fiber families evolve to similar values. The measured dispersions, that are shown in Fig. 4(d), reveal a different degree of alignment between both families at 60–80 µm of depth. Note that, at a depth of 60 µm and 65 µm, high dispersion values, ${b}_{2,3}\ge 0.2$, are measured. This is favored by a low fraction of fibers, $a\le 0.2$, contributing to fiber families 2 and 3. Below a depth of 80 µm, the dispersion of both fiber families is identical. The mean alignment of the fiber network, measured by the AI, continuously increases with depth (Fig. 4(e)). Contrary, the OI first increases until a depth of 75–80 µm and then decreases to a value of nearly zero at 105 µm. This is visually expressed by local fiber orientations covering almost the full angular half space (Fig. 4(e) and (f)). Below a depth of 80 µm, the color variety of local orientations reduces (Fig. 4(h)). At a depth of 105 µm, the large majority of fibers is visualized in green and blue/purple representing fiber orientations fluctuating around mean values of ${{\bar{\theta}}}_{1}=44.4^\circ$ and ${{\bar{\theta}}}_{2}=142.8^\circ$.

The collagen fiber network of soft tissue is in general not isotropic and expresses a preferred orientation^5,33,34^. In human skin, the preferred orientation is supposed to coincide with the main tension lines of skin, e.g. the Langer lines, which was confirmed ex-vivo^16,30^. However, scanning electron microscopy (SEM) and SHG ex-vivo experiments failed to support this model^35–38^.

Our results show that with the newly introduced FINE algorithm a direct analysis of a collagen network in-vivo is possible. The quantification algorithm allows to determine the number of fiber families without any previous assumptions about the underlying tissue. With increasing depth, mean fiber family orientations align perpendicular to each other such that no main orientation is expressed. The transition of the fiber network from overall dispersed fibers to aligned fibers at a depth of 80 µm is expressed by all parameters describing the collagen network. We associate this transition with the crossover from the papillary dermis to the reticular dermis, which is in line with Neerken et al., who measured the onset of the reticular dermis at a depth of $({95}\pm {10})$  µm at the temple^39^. The transition is additionally characterized by loose, thin collagen fibers in the papillary dermis that form a resilient network of thicker fibers in the reticular dermis^40^. The fiber status of the papillary dermis has been shown to be of main importance in skin aging^27,41^.

Furthermore, the FINE algorithm might be suitable for the classification of pathological deficiencies that impact the collagen fiber network like the Ehlers–Danlos syndrome^42^. It should be noted that our presented measurement serves as a proof of principle study and does not allow for a general conclusion, which would require a higher number of samples.

In conclusion, the FINE algorithm was found to be able to reliably quantify the fiber network by determining the number of fiber families, their mean orientations, amplitudes, dispersions as well as the orientation index, and the alignment index. The newly derived alignment index captures fiber family dependent information about the fiber network independently from the widely used averaged orientation index. Combined with in-vivo SHG microscopy of dermal collagen, we demonstrate a fully non-invasive and reliable algorithm to obtain meaningful insights into the composition of the dermal collagen fiber network. In general, the presented FINE algorithm is not limited to the application of dermal collagen. Potential applications might reach from different soft tissues to the quantification of any kind of fiber-reinforced material.

## Methods

Image processing, Monte-Carlo image generation and curve fitting was realized by using MATLAB^43^ in conjunction with the image processing toolbox and the curve fitting toolbox.

### Monte-Carlo fiber images

The generation of artificial, grayscale fiber images is described in detail by Witte *et al.*^23^. The orientation angles of fibers, which contribute to a certain fiber family are sampled from a semi-circular von-Mises distribution with mean orientation ${\bar{\theta}}$:

2 $$P\left(\theta ;{\bar{\theta}},k\right)=\frac{1}{\pi {I}_{0}\left(k\right)}{e}^{k\cos(2(\theta -{\bar{\theta}}))},$$

where the dispersion parameter $k$ defines the width of the distribution. A large value of $k$ describes a narrow angular distribution, which corresponds to a high degree of fiber alignment. The Monte-Carlo sampling is repeated for each defined fiber family. To achieve a true isotropic fiber distribution, fiber angles are equally distributed across the entire angular range $[{0}^\circ , {180}^\circ ]$. In addition, every fiber features a width and an aspect ratio, which defines its length. As fibers with a very small width were found to produce large errors^23^, we choose a minimum fiber width of 3 pixels. Similar to^20,23^ we set the maximum fiber width to 10 pixels. The aspect ratio is constrained to the interval^20,45^. Note that uniform fiber geometries (width, length) are used for each Monte-Carlo image. To account for different image qualities, we use a random noise factor which defines the amplitude of added speckle noise.

### Angular orientation distribution

We use a Fourier-based method (AF method), as proposed by Witte *et al.*^23^, to obtain the angular orientation distribution $I(\theta )$ of an image ${I}_{p}(x,y)$. Using the method, the power spectrum $P(u,v)$, which is defined as the square of the absolute value of the 2D discrete Fourier transform, is computed. First, coordinates are shifted such that low frequencies are located in the center of the power spectrum. In addition, the adaptive filter, which is based on the relative variance of the signal, is applied to the power spectrum. As shown by Witte *et al.*^23^, most accurate results are obtained by allowing relative variances smaller than 2.1%. To extract the angular orientation information, the signal of the filtered power spectrum is radially summed and normalized. The variance of the angular orientation distribution, $\Delta I(\theta )$, is obtained by propagating the variance of the image $\Delta {I}_{p}\left(x,y\right)=\sqrt{{I}_{p}\left(x,y\right)}$ to the Fourier domain^23^.

Similar to Witte *et al.*^23^, we employ the cumulative orientation distribution $C(\theta )$ (COD):

3 $$C\left(\theta \right)=\sum_{{\theta }^{{\prime}}=0^\circ }^{\theta }I(\theta ^{\prime})$$

Note that the angular orientation distribution is normalized with ${\sum }_{\theta =0 }^{180^\circ }I\left(\theta \right)=1$. The variance of the COD, $\Delta C(\theta )$, follows from propagating Eq. (3):

4 $$\Delta C\left(\theta \right)=\sqrt{\sum_{{\theta }^{{\prime}}=0^\circ }^{\theta }\Delta I{\left({\theta }^{{\prime}}\right)}^{2}}$$

Since the choice of the starting angle of computing the COD [Eq. (3)] is arbitrary, the variance of the COD, $\Delta C(\theta )$, has to be independent from $\theta$. Thus, we employ the variance as $\sigma =\mathrm{max}(\Delta C(\theta ))$. Note that within the FINE algorithm, σ is crucial for identifying additional, significant fiber families. For further information on the calculation of $\Delta C(\theta )$, please refer to^23^.

### Fit model

We model the COD by using a sigmoid function:

5 $${S}_{\text{circ}}\left(\theta \right)=A\cdot \left[S\left(\theta \right)+S\left(\theta +180^\circ \right)-S\left(180^\circ \right)+S\left(\theta -180^\circ \right)-S\left(-180^\circ \right)-S\left(0^\circ \right)\right]$$

6 $$\text{with}\quad S(\theta )=\frac{1}{1+{e}^{-b(\theta -{\bar{\theta}})}},$$

where ${\bar{\theta}}$ denotes the mean orientation and $b$ the steepness of the step, which is a measure of the fiber dispersion. The added terms take care of the semi-circularity of the angular orientation distribution and its characteristic in the cumulative orientation distribution. The factor A is chosen such that the employed sigmoid function [Eq. (5)] fulfills ${S}_{\text{circ}}\left({180}^\circ \right)={1}$ for all parameter ${\bar{\theta}}$ and $b$. This is given for:

7 $$A= \frac{1}{S\left({360}^{\circ }\right)-S(-{180}^{\circ })}.$$

To account for the contribution of multiple fiber families, a series of sigmoid functions is applied:

8 $${S}_{N}(\theta )=\left\{\begin{array}{ll}{S}_{\text{circ}} , & N=1\\ \sum_{i=1}^{N}{a}_{i}{S}_{\text{circ}}\left(\theta ;{b}_{i},{\bar{\theta }_{i}}\right), & N>1,\end{array}\right.$$

where $N$ denotes the number of fiber families. The $i$-th fiber family which exhibits a dispersion ${b}_{i}$ and a mean orientation ${{\bar{\theta}}}_{i}$ contributes with an amplitude of ${a}_{i}$. We relate the von-Mises dispersion parameter $k$ to the sigmoidal dispersion $b$ using a numerical transfer function $b(k)$, which is shown in Supplementary Fig. 4. For each value of $k$, we sample ${10}^{4}$ values from the respective von-Mises function. After calculating the cumulative distribution, we fit the sigmoid [Eq. (5)] to obtain the dispersion parameter $b$. Each datapoint of Supplementary Fig. 4 is calculated from averaging over 100 values. Best results are obtained by splitting the dataset into $k<{2}$ and $k\ge {2}$. We fit both datasets using a power function ${c}_{1}\cdot {k}^{{c}_{2}} +{c}_{3}$ with coefficients ${c}_{1}, {c}_{2}\,{\text{and}}\,{c}_{3}$.

Note that in Witte et al.^23^, a sigmoid model was proven to provide a more accurate representation of the mean orientation and dispersion of one fiber family compared to the classical von-Mises approach^12,22^. Especially the calculation of the dispersion parameter can be significantly improved using the sigmoidal approach. Further details on the comparison of both methods can be found in Witte *et al.*^23^.

### Isotropy criterion

In order to classify an unknown cumulative distribution function as isotropic prior to fit potential fiber families, a criterion similar to the approach of Schriefl *et al.*^22^ is used. Since an ideal cumulative distribution function of an isotropic distribution is a straight line with a slope of 1/180°, the initial fit of the FINE algorithm (Fig. 1) is used to evaluate the isotropy of the distribution. ${R}^{2}$ is used as parameter to measure the goodness of the fit. In total ${10}^{4}$ images with an isotropic fiber orientation distribution were created using the implemented Monte-Carlo method. 95% of the images were considered as isotropic for a threshold value of ${R}^{2}\ge 0.9916$. Even if an isotropic distribution fails the initial isotropy criterion, the fitted fiber family can be classified as isotropic retrospectively. We emphasize, that the isotropy criterion is not used to evaluate the significance of additional fiber families. Instead, the variance $\sigma =\Delta C(\theta )$ of the COD is evaluated and used in terms of a 3σ criterion in the FINE algorithm.

### Level of significance

The level of significance is controlled by multiplying the variance of the cumulative distribution function, $\Delta C(\theta )$, with a factor $n$. The right choice of $n$ is crucial in order to not over-interpret small fluctuations and still capture significant fiber families of the cumulative distribution. To find the factor which maximizes the accuracy to determine the number of identified fiber families, Monte-Carlo images that feature multiple fiber families (up to three) as well as images with a single, aligned family together with an isotropic family were included. To ensure an unambiguous differentiation of neighbored fiber families in the orientation distribution, a von-Mises dispersion of $k={10}\, (b=0.18)$ with a minimum distance of ${30}^\circ$ between neighbored families was chosen. Subsequently, the limits ${n}_{\min}$ and ${n}_{\max}$ defining the range of significance, in which the calculated number matches the defined number of fiber families, were determined. Each limit was calculated by repeatedly applying the implemented fit procedure on the cumulative distribution function while adapting the level of significance using a bisection algorithm. The algorithm was terminated as the difference between subsequent iterations was smaller than ${10}^{-{4}}$. The maximum accuracy of the algorithm was found at $n={3}$.

### Orientation index

In order to characterize the entire orientation distribution, we evaluate the orientation index (OI)^28^ and the novel alignment index (AI). The orientation index is based on the angular orientation distribution $I(\theta )$:

9 $${\text{OI}}=2\frac{{\int }_{{0}^{\circ}}^{{180}^{\circ }}I\left(\theta \right){\mathrm{cos}}^{2}\left(\theta -{{\bar{\theta}}}_{\text{mean}}\right) {\text{d}}\theta}{{\int }_{{0}^{\circ }}^{{180}^{\circ }}I\left(\theta \right)\text{ d}\theta}-1$$

A fully isotropic distribution results in a vanishing orientation index, whereas a full alignment of the fibers yields an OI of one. ${{\bar{\theta}}}_{\text{mean}}$ describes the overall mean orientation, which is determined from fitting a single sigmoid (${S}_{1}$) to the COD.

### Alignment index

The alignment index (AI) is defined in Eq. (1). Normalization constants ${b}_{\min}$ and ${b}_{\max}$ define the scale of the AI. The lower limit of the dispersion parameter is defined as ${b}_{\min}=0.016,$ which results from the transfer function $b\left(k\right)$ for $k\to {0}$. ${b}_{\max}$ corresponds to the maximum dispersion parameter at which the AI has a value of one. We consider a family as fully aligned if its dispersion parameter $b$ is equal to $0.26\, (k={20}).$ We therefore choose a value of ${b}_{\max}=0.26$. The defined scale is found to cover a large majority of the measured distributions without a saturation.

### Local fiber orientation

The local fiber orientation is achieved similarly to the fan-filter method proposed by McLean *et al.*^44^. In each pixel the angular distribution contributes to an orientation spectrum. The contribution of one specific angle $\theta ^{\prime}$ results from applying the inverse Fourier transform to the fan-filtered discrete Fourier transform of the image. The fan-filter is a wedge-shaped filter, that covers $\theta \in [{\theta }^{{\prime}}-\delta \theta ,{\theta }^{{\prime}}+\delta \theta ]$. Contrary to McLean et al., we define the fan-filter by computing the fraction of each pixel in the Fourier domain that is covered by the angle interval. Hence, we do not need to apply a Gaussian convolution to remove sharp filter edges that induce Gibbs artifacts. We use the fan-filter in the frequency domain with a subsequent inverse Fourier transformation for every angle $\theta \in [{0}^\circ ,{180}^\circ ]$ in ${1}^\circ$ steps with $\delta \theta =0.5^\circ$ to obtain local orientation spectra $I(x,y,\theta )$. Spectra are smoothed using a moving average filter with an angular span of ${7}^\circ$. We assign a color to each pixel based on the angle at maximum spectral intensity. In agreement with McLean *et al.*^44^, in non-fibrous areas of the image the amplitude of the RGB color is reduced using the relative intensity of the background-subtracted pixel. For background subtraction, the Fiji (ImageJ) build-in function subtract background with a rolling ball radius of 40 pixels is used^45^.

### Multi-photon microscopy

For collagen measurements we use a multi-photon microscope which was developed in collaboration with Jenlab GmbH (Jena, Germany)^46^. To measure the collagen-specific second-harmonic generation (SHG) signal, a femtosecond ti:sapphire laser (Mai Tai, Spectra-Physics, California, USA) adjusted to a wavelength of 820 nm is used in combination with a 410 nm band-pass filter (AQ 410/20 m-2P, Chroma Technology Corp., Bellows Falls, VT). The microscope was used at a scan time of 7 s, a constant mean illumination power of 50 mW, and a field of view that covers a 220 × 220 µm area at an image dimension of 512 × 512 pixels.

In order to measure the three-dimensional distribution of collagen, a 3D-stack of in total 10 SHG images is recorded at the forehead of a 53 years old male Caucasian. An image slice spacing of 5 µm was chosen. The initial depth of the stack resulted from the onset of visible collagen fibers which was at a depth of 60 µm. The maximum depth of 105 µm was limited by the decreasing image quality. Depth is measured relative to the skin surface which was recorded by measuring the two-photon autofluorescence signal of the uppermost skin layer using an excitation wavelength of 750 nm with a $({548}\pm {150})$ nm band pass filter (HQ 548/305 m-2P, Schott AG, Mainz, Germany).

This study was conducted according to the recommendations of the current version of the Declaration of Helsinki and the Guideline of the International Conference on Harmonization Good Clinical Practice, (ICH GCP). In addition, this study was approved and cleared by the institutional ethics review board (Beiersdorf AG, Hamburg, Germany). Written informed consent was obtained from the volunteer.

## Supplementary information

Supplementary information.
