# Supplementary material for: General method for classification of fiber families in fiber-reinforced materials: application to in-vivo human skin images
Source: Sci Rep. 2020 Jul 2;10:10888. doi: 10.1038/s41598-020-67632-z (PMC7331592; doi:10.1038/s41598-020-67632-z)
Supplement: Supplementary file 1 — Supplementary information. [file 41598_2020_67632_MOESM1_ESM.docx]

**Supplementary information**

**General Method for Classification of Fiber Families in Fiber-reinforced Materials - Application to in-vivo Human Skin Images**

Maximilian Witte^1,2^, Sören Jaspers^2^, Horst Wenck^2^, Michael Rübhausen^1^ and Frank Fischer^2 ,^*

1. Center for Free-Electron Laser Science (CFEL), University of Hamburg, Hamburg, 22607, Germany
2. Beiersdorf AG, Hamburg, 20245, Germany

- [Frank.Fischer@beiersdorf.com](mailto:Frank.Fischer@beiersdorf.com)

**Supplementary Figures**

| 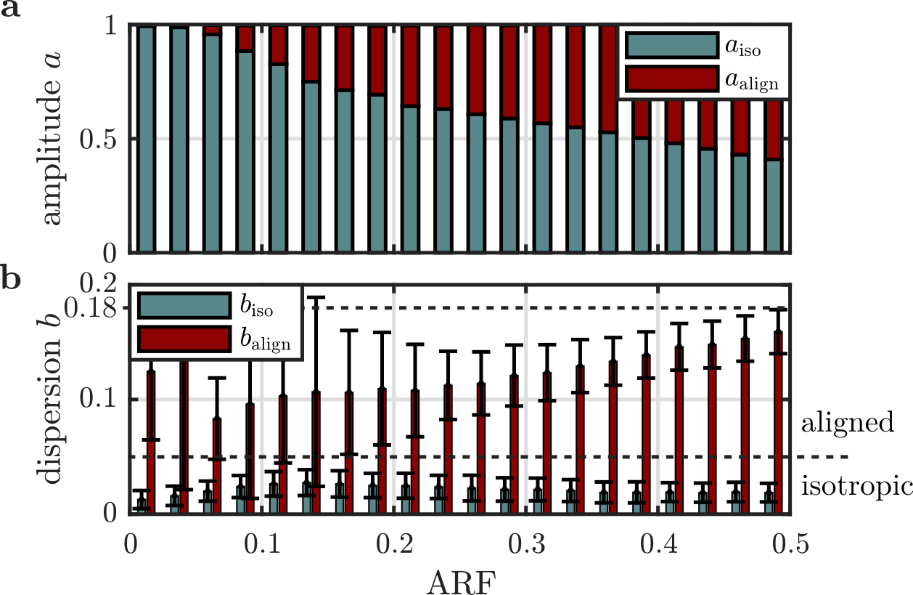 |
| --- |
| **Supplementary Figure 1.** Result of the fiber family fit algorithm as a function of the anisotropic ratio of fibers (ARF) of Monte-Carlo simulated fiber images.  **(a**) Mean amplitudes of the isotropic and the aligned fiber families that were identified by the algorithm. (**b**) Mean dispersions of the isotropic and the aligned fiber families that were identified by the algorithm. The isotropic threshold at $b=\text{0.05}$ as well as the predefined dispersion of the aligned fiber family are marked with dotted lines. Errorbars represent the standard deviation. |

| 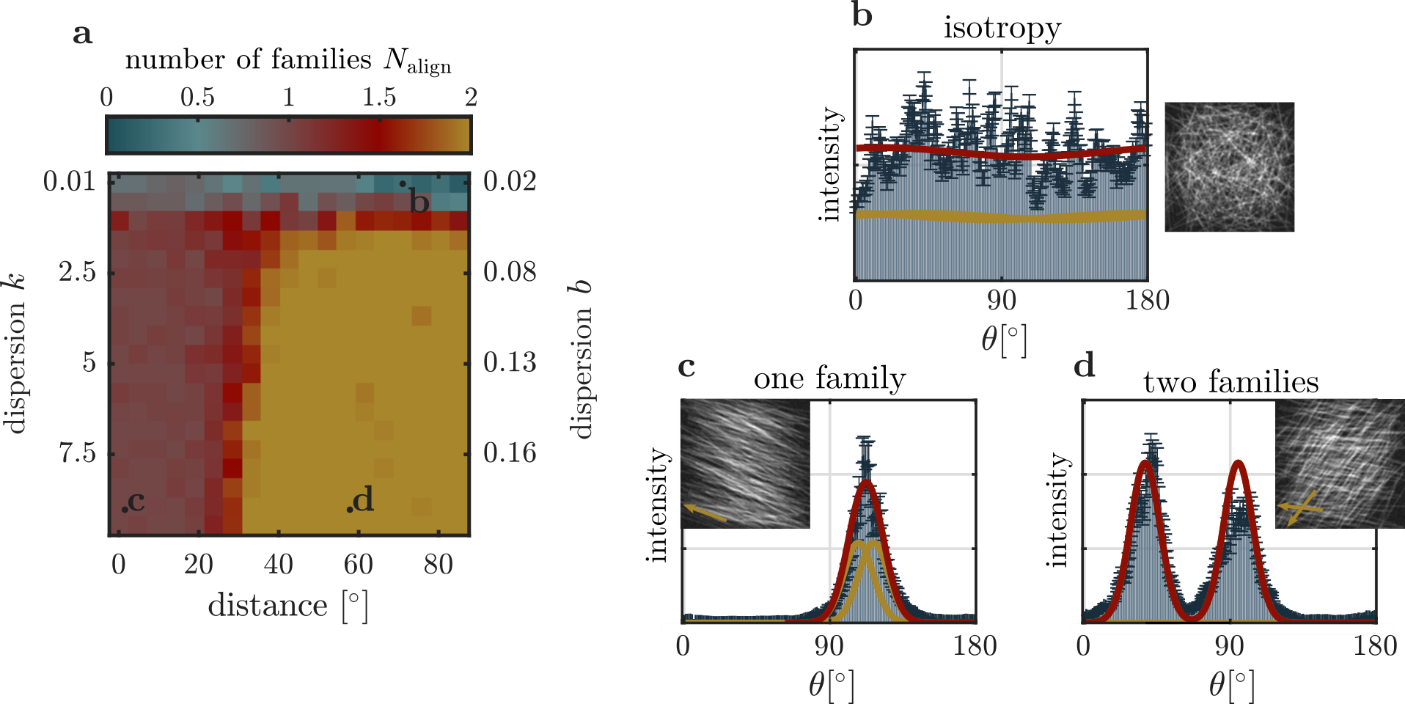 |
| --- |
| **Supplementary Figure 2.** Results of the fiber family fit algorithm of Monte-Carlo simulated images with two fiber families.  (**a**) The mean number of aligned fiber families as a function of their dispersions and the distance between their mean orientations. Exemplary angular distributions are given in (**b** - **d**). (**b**) Example of the calculated angular orientation distribution of two isotropic fiber families with a distance of 70°. The distribution of sampled fiber angles of both families is shown in yellow. The red curve represents the addition of both distributions. (**c**) Example of the angular orientation distribution of two highly aligned fiber families with a distance of 5°. The mean direction of both fiber families is indicated by arrows in the bottom left corner. (**d**) Example of the angular orientation distribution of two highly aligned fiber families with a distance of 60°. Note that the red curve overlaps the yellow curves perfectly. |

| 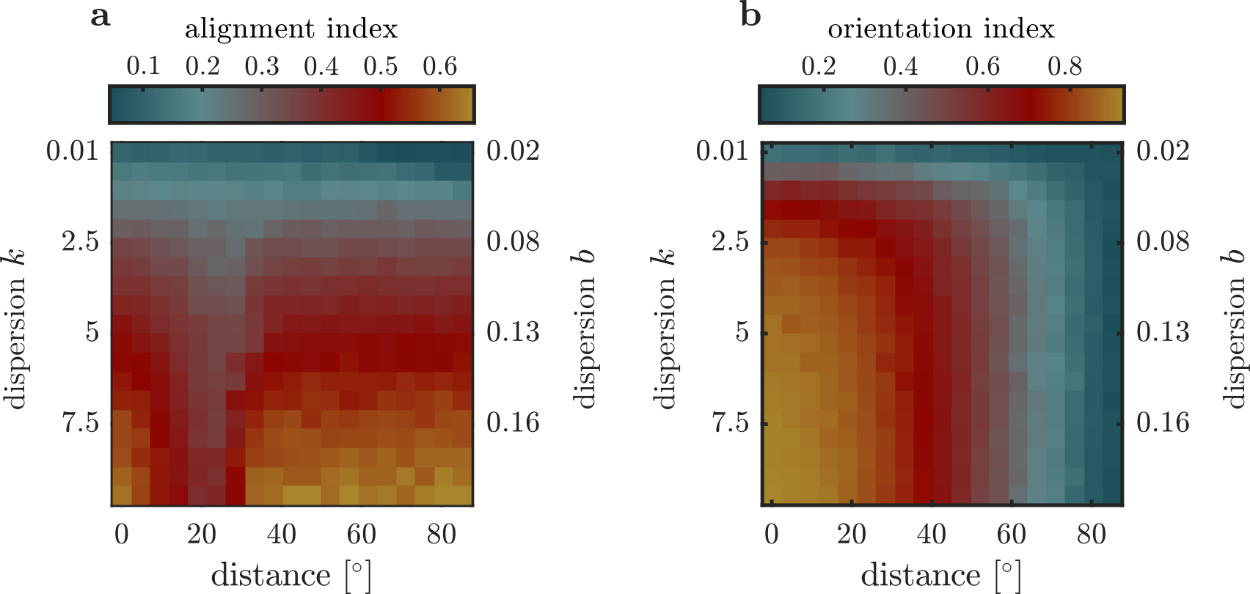 |
| --- |
| **Supplementary Figure 3.** Derived parameters of Monte-Carlo simulated images with two fiber families. The same dataset as in Supplementary Fig. 2 was used. (**a**) The alignment index as a function of their dispersions and the distance between their mean orientations. (**b**) The orientation index as a function of their dispersions and the distance between their mean orientations. |

| 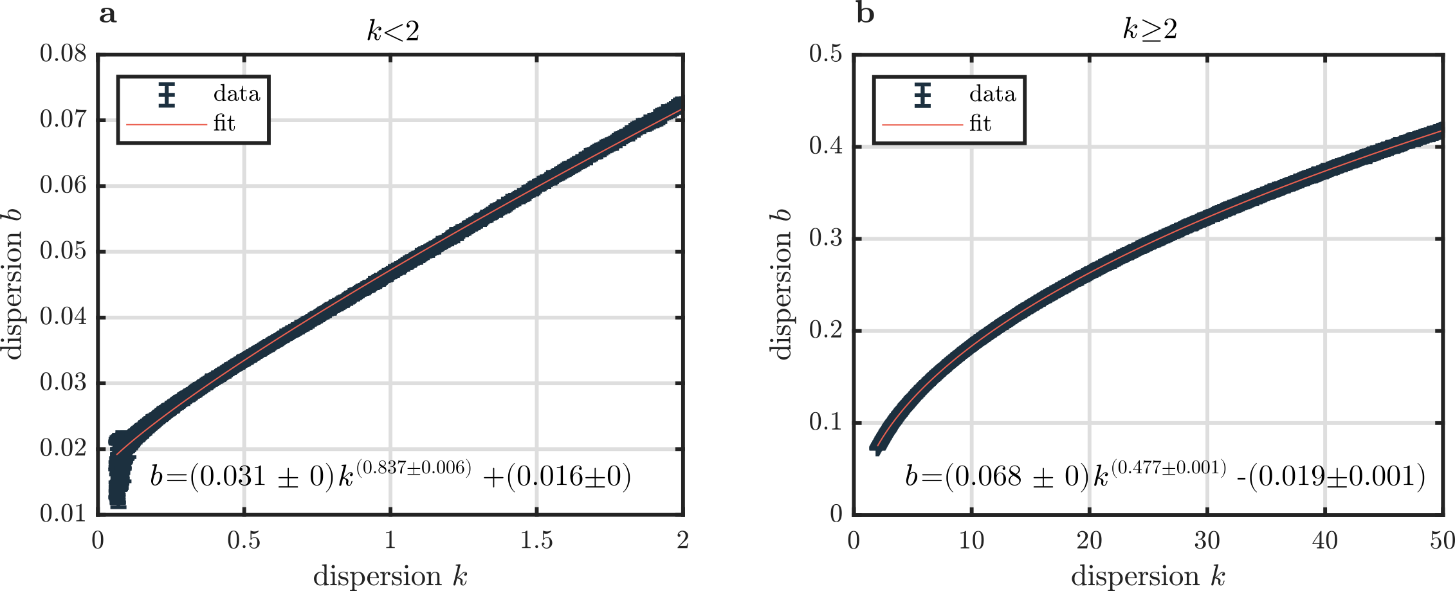 |
| --- |
| **Supplementary Figure 4.** Relationship between dispersion parameter b and k. (**a**) Sigmoidal dispersion b as a function of von-Mises dispersion k for $k<\text{2}$. (**b**) Sigmoidal dispersion b as a function of von-Mises dispersion $k$ for $k\geq\text{2}$. |
